# Proteomic analysis of protein carbonylation: a useful tool to unravel nanoparticle toxicity mechanisms

**DOI:** 10.1186/s12989-015-0108-2

**Published:** 2015-11-02

**Authors:** Marc D. Driessen, Sarah Mues, Antje Vennemann, Bryan Hellack, Anne Bannuscher, Vishalini Vimalakanthan, Christian Riebeling, Rainer Ossig, Martin Wiemann, Jürgen Schnekenburger, Thomas A. J. Kuhlbusch, Bernhard Renard, Andreas Luch, Andrea Haase

**Affiliations:** Department of Chemicals and Product Safety, German Federal Institute for Risk Assessment (BfR), Berlin, Germany; Biomedical Technology Center, Westfälische Wilhelms-University, Münster, Germany; IBE R&D gGmbH, Institute for Lung Health, Münster, Germany; Institute of Energy and Environmental Technology (IUTA) e.V., Air Quality & Sustainable Nanotechnology, Duisburg, Germany; Center for Nanointegration CENIDE, University of Duisburg-Essen, Duisburg, Germany; Robert-Koch-Institut (RKI), Junior Research Group Bioinformatics, Berlin, Germany

**Keywords:** Surface functionalization, Protein carbonylation, Oxidative stress, Silica nanoparticles, Zirconium oxide nanoparticles, Silver nanoparticles, Rat lung, ESR, DCFDA

## Abstract

**Background:**

Oxidative stress, a commonly used paradigm to explain nanoparticle (NP)-induced toxicity, results from an imbalance between reactive oxygen species (ROS) generation and detoxification. As one consequence, protein carbonyl levels may become enhanced. Thus, the qualitative and quantitative description of protein carbonylation may be used to characterize how biological systems respond to oxidative stress induced by NPs.

**Methods:**

We investigated a representative panel of 24 NPs including functionalized amorphous silica (6), zirconium dioxide (4), silver (4), titanium dioxide (3), zinc oxide (2), multiwalled carbon nanotubes (3), barium sulfate and boehmite. Surface reactivities of all NPs were studied in a cell-free system by electron spin resonance (ESR). NRK-52E cells were treated with all NPs, analyzed for viability (WST-1 assay) and intracellular ROS production (DCFDA assay). Carbonylated proteins were assessed by 1D and/or 2D immunoblotting and identified by matrix assisted laser desorption time-of-flight mass spectrometry (MALDI-TOF/TOF). In parallel, tissue homogenates from rat lungs intratracheally instilled with silver NPs were studied.

**Results:**

Eleven NPs induced elevated levels of carbonylated proteins. This was in good agreement with the surface reactivity of the NPs as obtained by ESR and the reduction in cell viability as assessed by WST-1 assay. By contrast, results obtained by DCFDA assay were deviating. Each NP induced an individual pattern of protein carbonyls on 2D immunoblots. Affected proteins comprised cytoskeletal components, proteins being involved in stress response, or cytoplasmic enzymes of central metabolic pathways such as glycolysis and gluconeogenesis. Furthermore, induction of carbonyls upon silver NP treatment was also verified in rat lung tissue homogenates.

**Conclusions:**

Analysis of protein carbonylation is a versatile and sensitive method to describe NP-induced oxidative stress and, therefore, can be used to identify NPs of concern. Furthermore, detailed information about compromised proteins may aid in classifying NPs according to their mode of action.

**Electronic supplementary material:**

The online version of this article (doi:10.1186/s12989-015-0108-2) contains supplementary material, which is available to authorized users.

## Background

The range of industrial processes and products taking advantage of nanotechnology has been growing rapidly in recent years. Current uses of nanoparticles (NPs) comprise e.g. the use of ZnO and TiO_2_ as UV protection agents in cosmetics and, due to their biocidal activity, silver NPs in packaging or medical devices, and ZrO_2_ and SiO_2_ as binders in ceramics and fillers in modern polymers. With this ever increasing diverse and common use of NPs it has become important to address concerns regarding possible adverse health effects. Currently, adaptation of test guidelines for NPs and debates on their validity for NPs are still ongoing [1]. In particular, fast and reliable toxicity screening methods are urgently needed as well as knowledge on the underlying toxic modes of action [2, 3]. Especially the latter aspect will foster the development of reliable alternative testing methods and may help to guide production of safe-by-design NPs. Furthermore, a more detailed knowledge of toxicity mechanisms is helpful for a regulatory prioritization and also for any successful grouping approach.

One prevailing paradigm explaining NP-mediated toxicity is the induction of oxidative stress [4–8]. Oxidative stress has been linked to various adverse outcomes such as inflammation, DNA damage, and general cytotoxicity [9]. It results from an imbalance between reactive oxygen species (ROS) generation and cellular antioxidants. Intracellular ROS are generated as regular byproduct of the respiratory chain and other oxygen consuming reactions [10]. Furthermore, ROS are induced in to cells by NPs in different ways [2, 11–17]. Among these, surface reactions driven by excitation of electrons via UV-light, Fenton-type reactions, catalytic chemistry at the NP surface, or via dissolved (metal) ions are implicated most prominently [2]. The types of ROS generated depend in particular on the NP and its chemical environment [18]. Various detection methods are available. Free radicals can be detected in vitro via electron spin resonance (ESR) spectroscopy using reagents called spin traps. These molecules form adducts to stabilize the radicals which then exhibit a paramagnetic resonance detectable by spectroscopy [18]. For instance, singlet oxygen and superoxide radicals can be detected using 1-hydroxy-3-carboxy-pyrrolidine (CPH), whereas 5,5-dimethylpyrroline N-oxide (DMPO) is sensitive to hydroxyl and superoxide radicals [18]. Furthermore NPs may induce ROS in cells due to other mechanisms as described below [11]. Xia and coworkers suggested that disruption of phagosomes leads to a first ROS peak, while a second increase stems from mitochondrial damage being part of apoptosis [11]. Thus a time and site-specific ROS generation along with specific byproducts may be expected. Moreover, it has been demonstrated that NPs may elicit a respiratory burst in professional phagocytes such as macrophages [14, 15], which use a NADPH-oxidase dependent defense mechanism primarily against viruses or bacteria together with expression of cytokines [12, 13]. In tissues or complex cell models, prolonged activation of cytokines and ROS may lead to oxidative damage and stress in surrounding cells, causing inflammation and potentially promoting DNA damage and tumorigenesis [19]. Finally, chronic depletion of cellular antioxidants such as glutathione renders cells vulnerable for oxidative stress [16] and stimuli by cytokines [17], possibly amplifying NP effects.

ROS detection in cells often uses fluorescent dye-based approaches, for example loading with dichlorofluorescein (DCFDA). However, these dyes when used in nanotoxicological studies may suffer from interferences with the NPs [6] and, albeit widely used, are prone to some risk of falsification. Thus, an alternative method to report on cellular ROS generation is highly needed. It is long been known that ROS cause oxidative modifications of cellular components, and especially proteins have often been described as predominant targets for oxidative modifications as they may scavenge up to 70 % of ROS [20, 21]. Thus, formation of protein carbonyls are among the most prevalent oxidative lesions of proteins [22]. Carbonylation is irreversible and usually results in an impairment or even loss of protein function, often associated with protein unfolding and aggregation. It may also be involved in signal transduction [23]. Different types of protein carbonyls (aldehydes and ketones) are formed either by peptide backbone fragmentation, side chain oxidation, or by a secondary reaction with oxidized cellular metabolites [24]. Analysis of oxidatively modified proteins in neurodegenerative diseases revealed that affected proteins are mainly involved in glucose metabolism, mitochondrial function, cellular motility/structural integrity, and protein degradation [25]. Various metabolic diseases [20, 26, 27], neurodegenerative diseases [25] as well as aging and age-related diseases occur along with elevated protein carbonyl levels [28, 29]. Furthermore several chemicals can enhance protein carbonylation. For instance in black tiger shrimp, the banned insecticide and acaricide endosulfan increased lipid peroxidation levels and protein carbonylation [30] as did the hepatotoxic and hepatotumorigenic fungicide propiconazole in mice [31] and ethanol exposure in rats [32]. Protein carbonyls are increasingly studied, e.g., in environmental toxicology where they are used as biomarkers of oxidative stress, as well as indicators of toxicological modes of actions [33–36] as usually the observed carbonylation pattern is dependent on the toxicant. Silver NPs were also shown to induce carbonyls in *Daphnia magna* [37].

Protein carbonyls can react with 2,4-dinitrophenylhydrazine (DNPH) and the resulting 2,4-dinitrophenylhydrazones can be detected with 2,4-dinitrophenyl specific antibodies in precipitates [38] or immunoblots [39, 40]. The latter approach has been used to detect protein carbonyls in obese mice [41], and humans [27]. With respect to NPs it has been shown that silver but not gold NPs induced protein carbonylation in THP-1 macrophages, primary neuronal cells [42, 43] and in a human colon epithelial cell line in a particle size dependent manner [44].

Especially the data on NP-treated cells suggest that analyzing protein carbonylation may be a useful tool for studying qualitatively and quantitatively the level of oxidative stress which had been acting upon NP-exposed cells or tissues. The derivatization of carbonyls with DNPH followed by immunoblotting is a specific and powerful technique, as it separates analytes and NPs and, therefore, is not expected to suffer from any NP interference.

The aim of the present study was to describe and compare the protein carbonyl pattern in NRK-52E cells subjected to a representative set of 24 NPs comprising amorphous silica (6 different types), zirconium dioxide (4 different types), silver (4 different types), titanium dioxide (3 different types), zinc oxide (2 different types), multiwalled carbon nanotubes (MWCNT, 3 different types), barium sulfate and boehmite (AlOOH). Results were evaluated and discussed with respect to chemical composition and different surface functionalizations. To investigate whether there is a relevance of NP treatment also in vivo we analyzed lung tissue of silver NP instilled rats in parallel. In this study we applied and compared different methods in parallel to the analysis of carbonylated proteins.

## Results

### Nanoparticle characterization

All nanoparticles (NPs) were dispersed in H_2_O and in complete cell culture medium (CCM; DMEM cell culture medium supplemented with 10 % fetal calf serum), the latter of which was the relevant biological test medium for the in vitro studies. About half of the NPs were dispersed by a stirring-based dispersion protocol (see Table 1) to preserve the functionalized surfaces. However, some of the NPs were hydrophobic and therefore difficult to disperse by stirring. Thus, we also included sonication-based dispersion methods for some of the NPs (see Table 1). We analyzed dispersion quality in water and CCM and also tested for stability of the dispersions in CCM over a time course of 24 h. As shown in Table 1 most NPs were well dispersed in H_2_O and CCM, and mostly dispersions were stable for up to 24 h.

**Table 1:** Basic NP characterization

		Coating/Stablilizer	Disp^a^	Size	DLS										Zeta (pH 7.4)
				TEM	H_2_O	CCM			CCM, 6 h			CCM, 24 h			
				mean [nm]	D50 [nm]	D50 [nm]			D50 [nm]			D50 [nm]			[mV]
SiO_2_	Unmod.	None	Stir	15	40	280	±	90	423	±	180	761	±	145	−39
	PEG	Polyethylene-glycol (MW 500 g/mol)	Stir	15	50	641	±	114	606	±	61	638	±	68	−26
	Amino	Aminopropyltrimethoxysilane	Stir	15	42	517	±	257	1171	±	865	1248	±	782	0
	Phosphate	TPMP	Stir	15	40	221	±	23	199	±	28	206	±	22	−42.9
	NM-200	None	US	50^b^	232	238	±	17	438	±	56	555	±	98	−47.5^b^
	NM-203	None	US	73.61^b^	159	319	±	51	468	±	29	705	±	408	−46.1^b^
ZrO_2_	Acrylate	Acrylate	Stir	9	9	925	±	260	1195	±	296	1161	±	245	−39
	PEG	Polyethylene-glycol (MW 500 g/mol)	Stir	9	27	265	±	420	252	±	8	261	±	22	−7.8
	Amino	Aminopropyltrimethoxysilane	Stir	10	315	644	±	163	657	±	166	815	±	217	3.9
	TODS	TODS	Stir	9	9	219	±	16	217	±	14	212	±	8	−6.5
Ag	50 PVP	Polyvinyl-pyrrolidone	Stir	97	123	226	±	22	214	±	20	196	±	10	−7
	200 PVP	Polyvinyl-pyrrolidone	Stir	134	408	271	±	36	264	±	37	272	±	41	−7
	50 Citrate	Citrate	Stir	20	35	131	±	3	125	±	4	128	±	5	−45
	NM-300k	Polyoxyethylene Glycerol Trioleate/Polyoxyethylene (20)/Tween 20	US	17^b^	71	65	±	1	81	±	10	77	±	17	n.a.
TiO_2_	NM-103 (rutile)	Polydimethyl siloxane	US	26^b^	299	315	±	63	312	±	32	364	±	62	n.a.
	NM-104 (rutile)	Polydimethyl siloxane	US	26^b^	244	268	±	30	283	±	10	281	±	28	n.a.
	NM-105 (15 % rutile/85 % anatase)	None	Stir	21	478	787	±	115	823	±	163	505	±	54	−17
MWCNT	NM-400	Not specified	US	11/846^b^	212^c^	209	±	23	259	±	38	253	±	14	n.a.
	NM-401	Not specified	US	67/4048^b^	762^c^	798	±	39	951	±	86	955	±	57	n.a.
	NM-402	Not specified	US	11/1372^b^	176^c^	191	±	11	166	±	9	174	±	19	n.a.
ZnO	NM-111	Triethoxycapryl silane	US	152^b^	345	285	±	17	282	±	11	304	±	14	n.a.
	NM-110	None	Stir	80	275	1020	±	182	901	±	142	759	±	253	20
	NM-110	None	US	65	436	482	±	19	474	±	8	449	±	33	n.a.
BaSO_4_	NM-220	None	Stir	32	350	353	±	24	335	±	20	449	±	136	−39
AlOOH		None	Stir	37	262	484	±	63	536	±	121	442	±	80	5

^a^Stir = dispersion by stirring, US = dispersion by sonication

^b^taken from JRC reports

^c^note that DLS is not suitable for measurement of MWNCT

### Nanoparticle surface reactivity measured by ESR

Next, we measured the surface reactivity of the NPs in a cell free system in H_2_O by ESR spectroscopy (Table 2) using CPH and DMPO as spin reagents. In total, 12 out of 23 NPs tested by ESR either had a significant surface reactivity with CPH or indicated fenton-like reactions occuring with DMPO. Applying the CPH spin probe we detected positive surface reactivity with SiO_2_ (unmodified, NM-200, NM-203), Ag 50 PVP, Ag 200 PVP, TiO_2_ NM-103 and ZnO (NM-110, NM-111). In addition, SiO_2_ (unmodified, Amino, Phosphate), TiO_2_ NM-105, MWCT NM-402 as well as ZnO (NM-110, NM-111) were found reactive in the presence of DMPO (Table 2).

**Table 2:** NP oxidative stress potential

		Cytotoxicity	Surface reactivity NP (NP free supernatant)			ROS – Activity		Protein carbonylation	
		IC50 (WST)	ESR	ESR	ESR	DCFDA-Assay		1D screen	2D test
		μg/ml (μg/cm^2^)	CPH/ dH2O	DMPO/dH2O	Overall	μg/ml (μg/cm^2^)	Highest treatment μg/mL (μg/cm^2^)	Relative	Spot number
SiO_2_	unmod.	weak (IC 50 not reached)	4 (0.88)	11 (6.3)	Positive	n.d.	34 (10)	Strong	74
	PEG	no cytotoxicity	1 (3.4)	11 (13)	Negative	n.d.	34 (10)	Negative	N.A.
	Amino	no cytotoxicity	0.57 (1.1)	21 (5.2)	Positive	n.d.	34 (10)	Medium	78
	Phosphate	no cytotoxicity	2.2 (1.2)	19 (5)	Positive	n.d.	34 (10)	Weak	78
	NM-200	no cytotoxicity	3.7	1.0	Positive	n.d.	170 (50)	Medium	86
	NM-203	week (IC 50 not reached)	3.3	1.1	Positive	n.d.	170 (50)	Medium	121
ZrO_2_	Acrylate	no cytotoxicity	1 (2)	3.6 (1.5)	Negative	n.d.	34 (10)	Negative	N.A.
	PEG	no cytotoxicity	1.5 (2.3)	1.7 (0.71)	Negative	n.d.	34 (10)	Negative	N.A.
	Amino	no cytotoxicity	0.95 (17)	3.5 (2)	Negative	n.d.	34 (10)	Negative	N.A.
	TODS	no cytotoxicity	0.54 (5.7)	0.94 (1.3)	Negative	n.d.	34 (10)	Negative	N.A.
Ag	50 PVP	1 (0.3)	45 (1)	0.4 (1)	Positive	n.d.	34 (10)	Strong	93
	200 PVP	no cytotoxicity	72 (1)	0.48 (1)	Positive	n.d.	34 (10)	Negative	N.A.
	50 Citrate	no cytotoxicity	N.A.	N.A.	N.A.	n.d.	34 (10)	Medium	44
	NM-300 k	12 (3.6)	1.4 (4.3)	0.5 (1.3)	Negative	n.d.	34 (10)	Medium	62
TiO_2_	NM-103	no cytotoxicity	3.2	1.0	Positive	n.d.	85 (25)	Negative	N.A.
	NM-104	no cytotoxicity	1.6	0.9	Negative	n.d.	85 (25)	Negative	N.A.
	NM-105	Strong	0.82	3	Positive	n.d.	34 (10)	Medium	57
MWCNT	NM-400	no cytotoxicity	2.1	1.2	Negative	34 (10)^a^	170 (50)	Negative	N.A.
	NM-401	no cytotoxicity	2.4	0.8	Negative	170 (50)^a^	170 (50)	Negative	N.A.
	NM-402	no cytotoxicity	1.1	3.4	Positive	34^a^ (10)	170 (50)	Negative	N.A.
ZnO	NM-111	20 (6)	2.8	3.5	Positive	n.d.	170 (50)	Strong	88
	NM-110 (stir)^b^	7.5 (2.2)	22	12	Positive	n.d.	170 (50)	Strong	68
	NM-110 (US)^b^	12.5 (3.7)	3.4	2.8	Positive	n.d.	170 (50)	Strong	80
BaSO_4_	NM-220	no cytotoxicity	2	2	Negative	N.A.	N.A.	Negative	N.A.
AlOOH		no cytotoxicity	2.3	1.3	Negative	N.A.	N.A.	Negative	N.A.

Values in brackets for ESR refer to NP-depleted supernatant, if applicable. Protein carbonylation strong, medium, and weak was assigned according to 1D immunoblots.

Data labeled with a) are derived from Farcal et al. [71]

^b^refers to dispersion method used (stir: stirring or US: ultrasonication)

### Cell viability after NP treatment

All NPs were tested for cytotoxic effects on an immortalized rat kidney epithelial cell line, NRK-52E cells using NP concentrations of 0–100 μg/mL (0–30 μg/cm^2^) in a WST-1 assay (Table 2). This cell model has been chosen as it appeared highly sensitive with respect to the development of oxidative stress after NP treatment in pilot studies and closely resembles differentiated tissue in contrast to many dedifferentiated tumor cell lines. Using this dose range four NP treatments reached an IC_50_, namely ZnO NM-110 and NM-111, Ag 50 PVP and Ag NM-300 k (Table 2). Interestingly, the IC_50_ of NM-110 differed when dispersed by stirring (7.5 μg/mL = 2.2 μg/cm^2^) or sonication (12.5 μg/mL = 3.7 μg/cm^2^). TiO2 NM-105 was cytotoxic as well. SiO_2_ unmodified and SiO_2_ NM-203 were slightly toxic and did not reach an IC_50_ in the tested concentration range.

### ROS generation by DCFDA assay

Three out of 22 NPs that were tested by the DCFDA assay induced ROS in NRK-52E cells, namely the three MWCNTs. NM-400 and NM-402 showed an about 1.4 fold increase in fluorescence at a concentration of 34 μg/mL (10 μg/cm^2^), while NM-401 showed a similar increase only at 170 μg/mL (50 μg/cm^2^). However, it should be noted that due to interferences in the read-out some NPs (SiO_2_ unmodified/PEG/Amino/Phosphate, all ZrO_2_; Ag 50 PVP/Citrate, Ag 200 PVP, Ag NM-300, TiO_2_ NM-105) could be tested only up to concentrations of 34 μg/mL (10 μg/cm^2^).

### Protein carbonylation by 1D immunoblotting

To analyze protein carbonylation we treated NRK-52E cells with increasing NP concentrations of up to 100 μg/ml (30 μg/cm^2^). In total 11 out of 24 NPs were found to induce protein carbonylation (Table 2, Additional file 1: Figure S2). Particularly high reactivity was detected for SiO_2_ unmodified, Ag 50 PVP, and the ZnO variants. SiO_2_ Amino, Ag 50 Citrate, TiO_2_ NM-105, SiO_2_ NM-200, SiO_2_ NM-203, and Ag NM-300 K were also positive, albeit with lower reactivity. A weak reactivity was detected with SiO_2_ Phosphate. Two NPs, i.e. ZrO_2_ Amino and SiO_2_ PEG, showed a weak response, but this could be attributed to the supernatant controls, rendering the results unspecific and both NPs were considered negative in Table 2. The NP free supernatant controls were obtained after prolonged ultracentrifugation and should mainly contain the dispersant and stabilizers. All other NPs induced no protein carbonyls and were considered negative. Additional 1: Figure S2 depicts all immunoblots for the positive NPs, and Additional file 1: Figure S3 for the negative NP.

### Comparison of oxidative stress assessment

Comparing DCFDA and ESR measurements we found an agreement for only 8 out of 22 NP treatments (Table 2). In most cases these NPs were completely negative in both assays. In total we observed an overlap of 36 % (Table 3). Often, NPs were found positive in the ESR assay, but DCFDA results remained negative. We found a similar overlap between DCFDA and the carbonyl assay (8 of 22 NPs, equals 36 %), here no positive overlaps occurred. All NPs that gave a positive result in the carbonylation assay did not induce ROS measured by DCFDA. However, when comparing the carbonylation assay and ESR measurements we observed an excellent overlap. For 19 out of 23 NPs we found an agreement between the carbonyl and the ESR results, i.e. an overlap of 83 % (Tables 2 and 3). Interestingly, in most cases (20 out of 24 NPs) we also found an excellent correlation between results of the protein carbonyl assay and cell viability (Table 2), i.e. 83 % overlap.

**Table 3:** Comparison of oxidative stress assessments

		ESR		Conformity
		+	-	
Carbonylation (23 NPs)^a^	+	9	1	19/ 23 (83 %)
	-	3	10	
DCFDA (21 NPs)^a,b^	+	1	2	8/ 21 (38 %)
	-	11	7	

^a^Ag 50 Citrate not analyzed by ESR

^b^BaSO_4_ and AlOOH not analyzed in DCFDA

### Each NP induces a unique pattern of protein carbonyls in 2D immunoblots

**Fig. 1:**
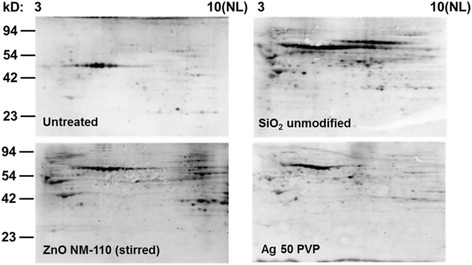
Protein carbonylation analyzed via 2D immunoblot. NRK-52E cells were treated for 6 h with 10 μg/ml of the indicated NP in at least 3 independent biological repeats. Carbonyls were detected using an anti-DNP antibody after coupling to 2,4-Dinitrophenylhydrazine and visualized by ECL. Representative blots are shown

**Fig. 2:**
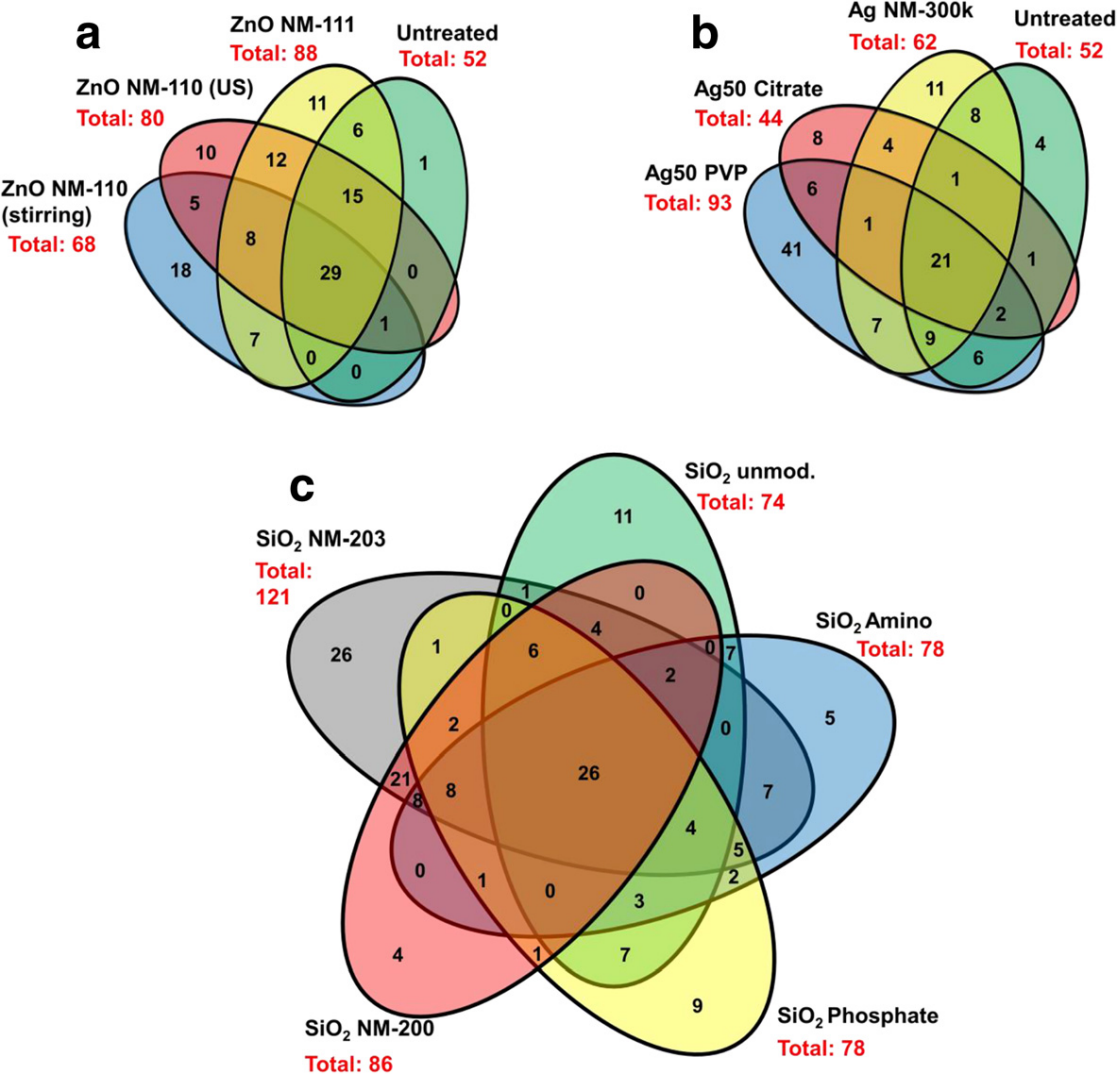
Venn diagrams. Similarities and differences in the spot pattern of protein carbonylation in the different treatments groups are visualized in Venn diagrams. Depicted are results for ZnO (**a**), nanosilver (**b**) and SiO_2_ variants (**c**). The sum of all numbers in one oval equals the total number of carbonylated spots for that respective NP. Numbers given in an overlap of two or more ovals represent the number of spots shared by the respective NPs

All NPs that were tested positive in the 1D screening assay were subjected to a more detailed analysis of the carbonylation patterns using 2D immunoblots. In total, we tested 11 different kinds of NPs: SiO_2_ (unmodified, Amino, Phosphate, NM-200, NM-203), Ag 50 PVP Ag 50 Citrate, Ag NM-300 K, TiO_2_ NM-105, ZnO NM-111 and ZnO NM-110 (note: NM-110 was tested with two different dispersion protocols). Figure 1 shows the 2D immunoblotting results for control (untreated) cells and cells treated with SiO_2_ unmodified, Ag 50 PVP, and ZnO NM-110. All other results are given in Additional file 1: Figure S4. In total we detected 202 carbonylated protein spots, out of which 55 % could be identified by mass spectrometry. In untreated control cells we found 52 protein spots carbonylated, which were also detected in most of the NP-treated samples. Each NP induced a unique pattern of carbonylated proteins. For a few NPs the spot pattern was similar to controls, although not identical. These NPs had a similar number of carbonylated proteins when compared to the control but displayed enhanced signal intensities in several of the carbonylated proteins, thus indicating a higher level of carbonylation for the respective proteins. We also analyzed the overlaps in the carbonylation pattern for all NPs (see Venn diagrams in Fig. 2). For instance, SiO_2_ NPs share 79 to 95 % of their respective carbonylated protein spots. Interestingly we could detect differences for ZnO NM-110, depending on the dispersion protocol used. Clearly, the stirring-based protocol resulted in a lower number of protein carbonyls (68 spots) compared to the sonication-based protocol (80 spots), 48 from these were found in common.

### Proteins involved in glycolysis, ATP synthesis and cell integrity/motility are carbonylated after NP treatment

From the total of 202 carbonyl spots 112 proteins (55 %) were successfully identified by mass spectrometry (Additional file 1: Table S1 and Additional file 1: Figure S5). Identification of some spots was impeded by very low protein amounts despite strong carbonyl immunoblot signals. In other cases, identification was hampered by very strong signals in immunoblots covering several neighboring protein spots in the corresponding duplicate gel. In these cases we excised all possible protein spots and these proteins were labeled “multiple” in Table S1.

**Fig. 3:**
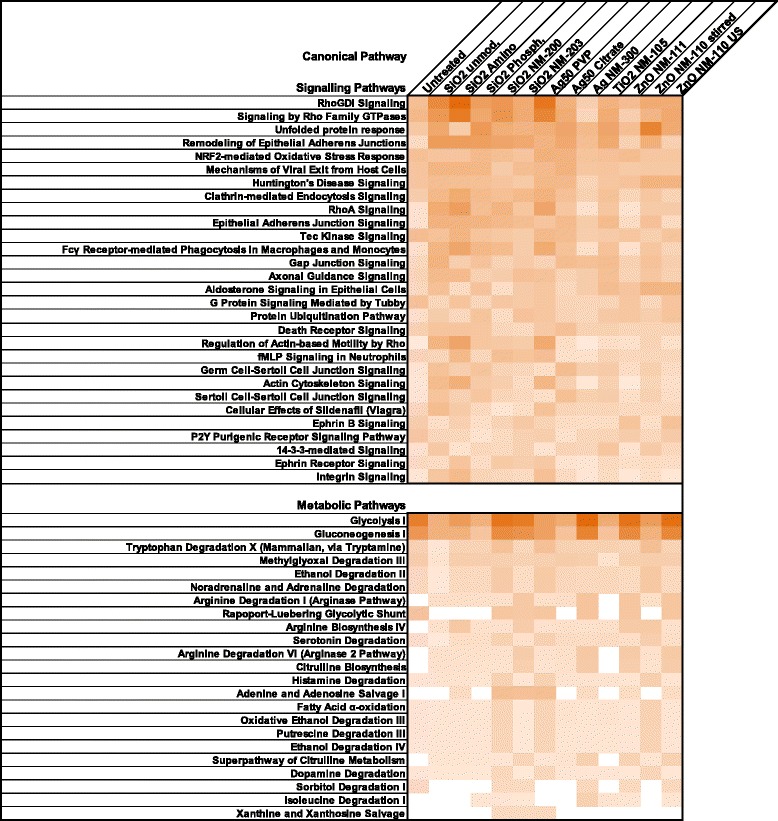
IPA Analysis of protein carbonylation in NRK-52E cells. Pathways are listed with decreasing probability of being affected by NP treatment. Color intensity reflects the number of carbonylated proteins belonging to the respective pathways. Signaling pathways are listed in the upper panel. Metabolic pathways are listed in the lower panel

**Fig. 4:**
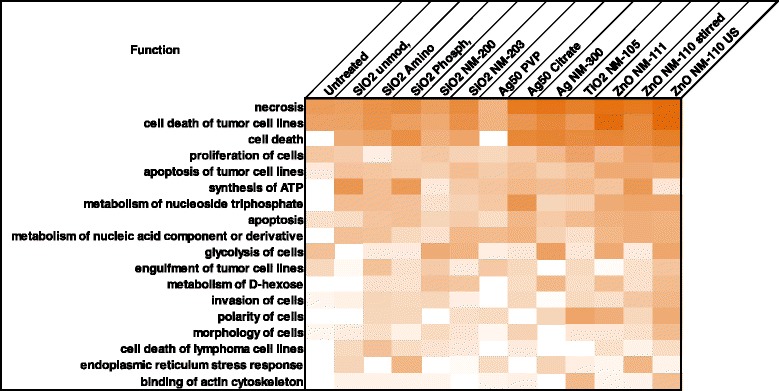
IPA Analysis of affected cellular functions. Cellular functions are listed with decreasing probability of being affected by carbonylation. Color intensity reflects the number of carbonylated proteins belonging to the respective pathways

The majority of carbonylated proteins comprised cytoskeletal proteins, proteins being involved in the cellular stress response, or enzymes of central metabolic pathways such as glycolysis or gluconeogenesis. To analyze affected signalling pathways in more detail we used Ingenuity pathway analysis (IPA). IPA confirmed that 8 out of 10 cytoplasmic enzymes involved in glycolysis and gluconeogenesis were carbonylated after NP treatment. Proteins involved in Rho signaling, the unfolded protein response, actin-based signaling, integrin signaling, and clathrin-mediated endocytosis appeared carbonylated as well. The complete list of assigned pathways is given in Fig. 3. Figure 4 shows an IPA of possibly affected cellular functions. Thus, elevated carbonylation of proteins is likely to be correlated with increased necrosis and cell death and impaired glycolysis and ATP synthesis.

### Principal component and hierarchical cluster analysis

Based on the 2D immunoblot data, i.e. based on the spot numbers and median intensities, we performed a statistical analysis (principal component and hierarchical cluster analysis) in order to analyze similarities between different treatments. These powerful tools can represent large amount of data in a concise way and identify possible clusters, which importantly could indicate a similar mode of action for the respective NPs.

**Fig. 5:**
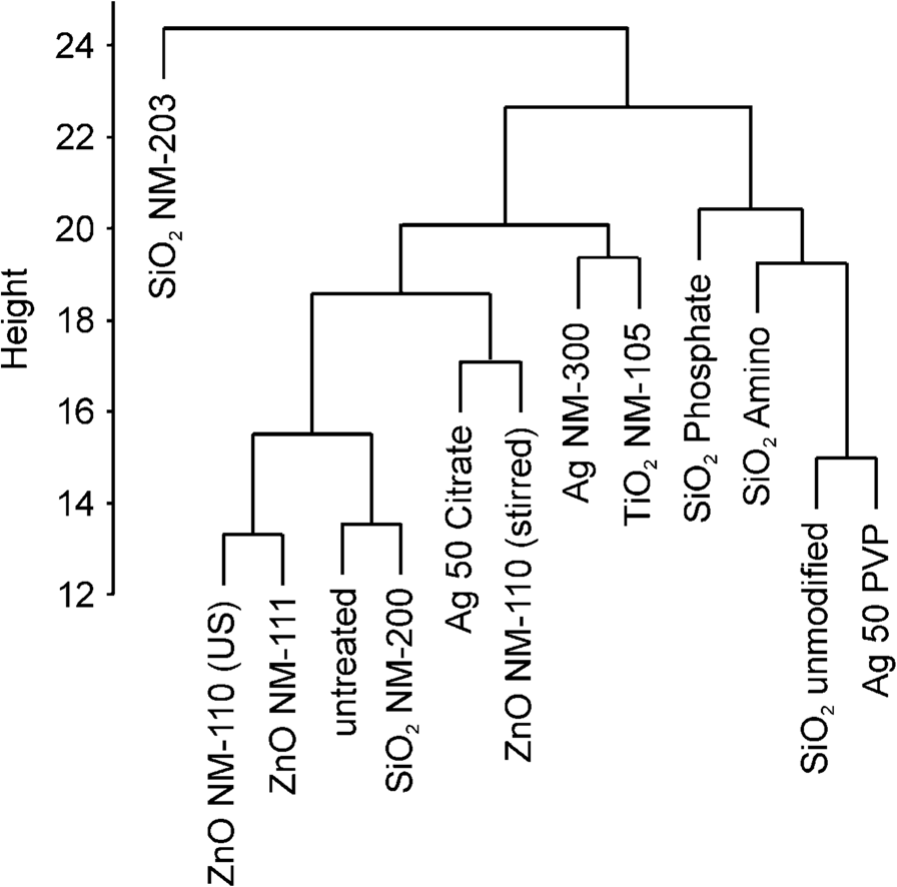
Hierarchical clustering analysis (HCA). HCA was performed using the median intensities of spots determined by image analysis of 2D immunoblots with Delta 2D software using a Euclidian complete linkage algorithm in R

Hierarchical cluster analysis (HCA) in this case compares the differences in normalized spot intensities among all data, thereby revealing clusters of nearest neighbors. We used a Euclidian complete linkage algorithm for the HCA (Fig. 5) which finds compact clusters of approximately equal diameters. We identified SiO_2_ NM-200 as being most similar to control cells. ZnO NM-110 and ZnO NM-111 were grouped together, indicating a similar mode of action. ZnO NM-110 (stirring protocol) was allocated to a different group, more similar to Ag 50 Citrate, but not together with ZnO (NM-110 and NM-111, ultrasonication-based protocol), again indicating a difference in mode of action based on the dispersion protocol. Interestingly SiO_2_ unmodified and Ag 50 PVP were grouped together as well, indicating a similar mode of action with respect to protein carbonylation.

**Fig. 6:**
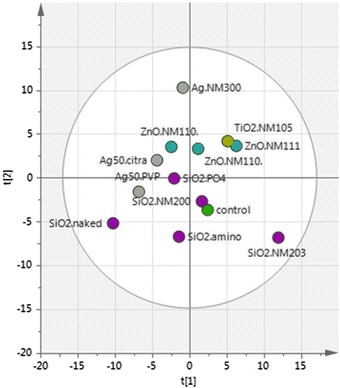
Principal component analysis (PCA). PCA was performed based on 5 main principal components using the median intensities of spots determined by image analysis of 2D immunoblots by Delta 2days software. Separation in the first two principal components is shown

Principal component analysis (PCA) finds the largest possible variability in the normalized data. Each subsequent principal component (PC) describes the largest variance in a direction orthogonally to the previous PC; thereby each PC is linearly uncorrelated. Plotting of the PCs against each other may then reveal groups of lower variance, i.e. they are similar in the parameters that contribute to the variance covered by the plotted PCs. Applying PCA with five PCs we could explain 65 % of the data variance. Figure 6 depicts the first two PCs covering 34 % of the data variance. Here we found SiO_2_ NM-200, SiO_2_ Amino and SiO_2_ Phosphate located closest to the untreated controls. SiO_2_ NM-203 was an outlier probably because it exhibits the highest number of spots (*c.f.* Fig. 2) albeit all with weak intensity. The ZnO NPs, TiO_2_, and Ag 50 Citrate grouped together around the axis of PC1 in the positive half of PC2, all of which display medium to strong carbonylation. SiO_2_ unmodified and Ag 50 PVP found in the negative half of PC1 and PC2, both very strong inducers of carbonylation and they were clearly separated from the other NPs. Overall analysis with PCA was in good agreement to results from HCA.

### Carbonylation in rat lung tissues after intratracheal instillation of silver NPs

**Fig. 7:**
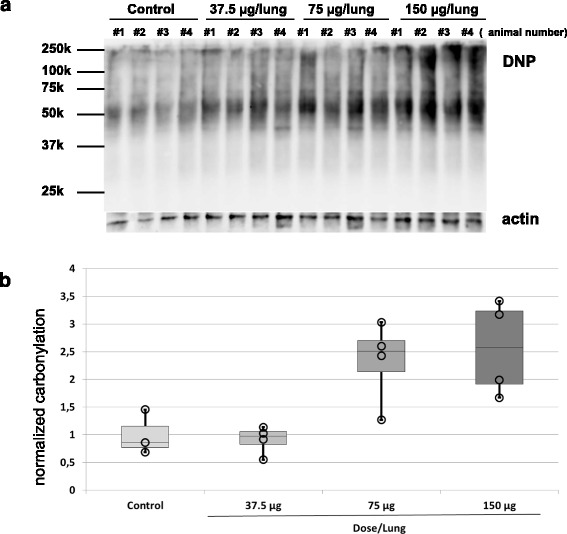
Elevated protein carbonylation in tissue lysates of rat lungs. **a** Tissue fragments from the left lungs of rats harvested 3 weeks after intratracheal instillation of Ag 50 PVP NP were analyzed for carbonylated proteins by 1D immune blotting. Actin served as a loading control. Control animals received 0.5 ml of vehicle (0.9 % NaCl). **b** Densitometric analysis of the immunoblot depicted in A). DNPH signals were summarized and adjusted to the actin signal. Animal #2 was excluded from analysis of the controls as the actin signals revealed that significantly less protein has been loaded compared to the other lanes

To analyze whether carbonylation upon Ag 50 PVP treatment also occurs in vivo we analyzed lysates from rat lung tissue blocks isolated 21 days after intratracheal instillation of Ag 50 PVP (37.5 μg, 75 μg, 150 μg, per lung). Figure 7 shows that the overall level of protein carbonylation as detected in 1D immunoblots was increased in a dose-dependent manner upon treatments with Ag 50 PVP. Compared to controls robust signals were especially obtained for high molecular weight proteins upon instillation of doses of 150 μg/rat lung.

## Discussion

### Analysis of protein carbonyls and ESR prove useful to analyze oxidative stress induced by NPs

In this study we compared three different approaches to analyze the oxidative stress potential inferred by a set of 24 NPs. Overall, ESR and DCFDA results were in agreement for 38 % of all NPs. Comparing the results of the 1D immunoblot carbonylation assay with ESR measurements, we found a much better overlap and results were in good agreement for 83 % of the NPs tested. Nearly all NPs, except for Ag NM-300 k, which induced protein carbonyls, were also tested positive by ESR. A few NPs (Ag 200 PVP, TiO_2_ NM-103, MWCNT NM-402) were tested positive in ESR but were negative in the carbonyl assay. Thus, surface reactivity, as indicated by either one of the two ESR reagents, appeared to be contributing to oxidative stress induction in cells, measurable as protein carbonylation. Since the results were not in perfect agreement for all cases, other aspects seem important as well (see below). Nevertheless, we conclude that ESR is well suited to analyze the biological relevant surface reactivity of NPs. However, it appears important to combine ESR results obtained with different reagents to fully describe biological ROS formation. It should be underlined that ESR analysis of NPs as produced (i.e. irrespective of changes that may occur in biological surroundings) appears to predict at least in part carbonylation of cytoplasmic proteins as a biological endpoint. This is an intriguing finding, as all particles were likely surrounded by a protein corona whose influence on direct oxidative processes remains elusive and requires further study.

Investigating oxidative stress of NP-treated cells by analyzing carbonylated proteins has some general advances. First, it circumvents all interferences with dyes or assay systems, as observed here for the DCFDA assay, in which the range of possible test concentrations was limited [5]. Secondly, by applying 2D-based proteome analysis it is possible to obtain detailed information on affected proteins and signaling pathways and these data can also be used to gain insight into the subcellular localization of oxidative processes. Thirdly, compared to the DCFDA assay, protein carbonyl analysis appears to be much more sensitive. Finally, analysis of protein carbonylation is also possible in NP-treated tissues after in vivo testing. The approach therefore appears useful to compare effects of NP treatment in vitro and in vivo in more detail, which is urgently required to further refine the conditions of in vitro testing.

### Analysis of protein carbonylation

All NPs that were tested positive in the 1D carbonyl screening assay were subsequently tested in a 2D-based approach in more detail. When comparing the results from the 2D approach two aspects need to be considered. One is the absolute number of carbonyl spots. For several NPs we observed an increased number of carbonylated protein species upon treatment. The second aspect to consider is the spot intensity. For a few NPs we did not detect much increase in the absolute spot number compared to untreated control cells but we detected increased spot intensities for several carbonylated protein species. Finally both kinds of information (i.e. spot number and spot intensity) need to be combined to obtain information on the biological activity. For instance, for SiO_2_ NM-203 elicited the highest number of carbonylated protein species (121 spots) but all of them showed moderate intensities on a quantitative scale such that overall carbonylation activity of SiO_2_ NM-203 is only medium (Table 2).

Carbonylation in untreated samples may be explained by a low amount of ROS that are produced in cells as a result of leakage from the mitochondrial electron transport chain. Moreover, it has repeatedly been reported that tumors exhibit a higher level of carbonylation compared to healthy tissue, which also holds true for cell lines (which are often derived from tumors) compared to e.g. primary cells [20]. In control cells, we mostly found carbonylation of proteins that are involved in stress responses such as chaperones, but also in proteins of the cytoskeleton or enzymes of the glycolysis. Although each type of NP induced a characteristic pattern of carbonylated protein spots, a quite significant overlap occurred which may reflect physico-chemical similarities of NPs.

#### Cytoskeletal proteins

Cytoskeletal proteins have often been identified as a main target of carbonylation [45] and this effect is not specific for NP. For instance, carbonylation of actin could be detected in vitro after treatment of cells with known toxicants, e.g. acrolein, hypochlorite, or chloramines. Mussels exposed to environmental pollutants showed increased actin carbonylation [46]. Furthermore, carbonylation of actin has been frequently linked to aging and also to several diseases, in particular neurodegenerative diseases like Alzheimer's Disease (AD), but also to heart failure, tumor growth, or prolonged inflammatory conditions [45]. In general, carbonylation of actin results in unfolding of actin monomers, depolymerization of actin strands, and aggregation of carbonylated actin. Since carbonylation of actin is also detected in untreated control cells it may be considered a ROS scavenger molecule under normal conditions.

However, we found excessive actin carbonylation in particular for SiO_2_ unmodified and Ag 50 PVP particles. Somewhat lower actin carbonylation was detected after treatment with SiO_2_ Amino/Phosphate/NM-200. A lesser degree of actin carbonylation was detected after treatment of the cells with Ag 50 Citrate and ZnO NM-110. In a similar manner tubulin carbonylation can be discussed. Actin and tubulin carbonylation are often detected together [34, 47].

#### Glycolysis / gluconeogenesis

Enzymes involved in glycolysis/gluconeogenesis are also often reported to be carbonylated. We identified up to 8 out of 10 glycolytic proteins carbonylated. Carbonylation of glycolytic enzymes can substantially decrease glycolysis turnover as shown in HL60 cells after treatment with etoposide (VP16) [48]. It has been speculated that impairment of glycolysis can put cells in an inactive state [48]. Depleting cellular ATP levels as a result of decreased glycolysis could lead to the inhibition of apoptosis [48]. In our study we found two glycolytic enzymes carbonylated in control cells, triosephosphate isomerase and glyceraldehyde-3-phosphate dehydrogenase. SiO_2_ NM-203 induced carbonylation of 8 out of 10 glycolytic enzymes. SiO_2_ phosphate, SiO_2_ NM-200 and Ag 50 PVP induced carbonylation of glycolytic enzymes as well. In summary, nearly all tested NPs augmented the carbonylation of glycolytic enzymes to various extents. However, there was no obvious link between toxicity and carbonylation of glycolytic enzymes. England et al. [48] suggested that carbonylation of glycolytic enzymes rather serves as protective mechanism which decreases the level of apoptosis. This is in line with the results observed for SiO_2_ NM-203, which showed no cytotoxicity despite a high level of carbonylation of glycolytic enzymes.

#### Other pathways

Several other pathways were found to contain carbonylated proteins after NP treatment. In particular, we found proteins of the unfolded protein response pathway carbonylated, especially chaperones or disulfide isomerases. This pathway was strongly affected by SiO_2_ unmodified/Phosphate and ZnO NM-110 (stirring). Furthermore, proteins of the Rho signaling pathway were found carbonylated after NP treatment, especially after treatment with SiO_2_ unmodified/Amino/NM-200 and Ag 50 PVP. The Rho signaling pathway is particularly important for cell migration, cell adhesion, cell polarity, changes in the actin cytoskeleton, and intracellular transport processes by vesicles. Considering that actin was modified by carbonylation as well, cell motility and cellular transport processes may be impaired by carbonylation. Our data suggest a good correlation between NP-induced carbonylation of proteins of the Rho signaling pathway (together with proteins of cytoskeleton/cell motility), and NP toxicity. At present, no information is available for membrane proteins associated with cellular organelles as they are hardly detectable in the 2D gels.

### Influence of NP’s chemical composition and surface modification on oxidative stress

Earlier studies have demonstrated that the capacity of a NP for inducing oxidative stress depends on its size (mostly due to the higher surface area/mass ratio) [49, 50] and surface modification [51, 52]. For silica NPs it has been reported that they can induce oxidative stress in vitro [52] and in vivo [53]. Yoshida et al. showed that surface modification of uncoated 70 nm silica particles by amination or carboxylation decreased cytotoxicity, DNA damage, and intracellular ROS generation in HaCaT and TLR-1 cells [52]. Similarly, Imai et al. reported a decreased inhibition of CYP3A4 activity by 30 and 70 nm silica NPs in HepG2 cells after surface carboxylation [54].

In our study we confirm that unmodified silica NPs cause stronger effects with respect to carbonylation than any of the surface modified SiO_2_ NPs. Yet, differences between the modified SiO_2_ Amino or SiO_2_ Phosphate and the larger, unmodified SiO_2_ NM-200 and SiO_2_ NM-203 are sparse compared to the strong effects of unmodified SiO_2_.

Also for silver NPs, oxidative stress has been reported in vivo [55] and in vitro [42]. Comparing the results between the different types of silver that have been tested in our study we found both a size-dependent effect (i.e. compare 50 and 200 nm PVP coated silver) and a coating-dependent effect (i.e. compare 50 nm silver citrate vs PVP coating). A size-dependent induction of oxidative stress has previously been demonstrated for nanosilver. Carlson et al. found an up to 10-fold increase of ROS activity (via DCFDA assay) in a rat alveolar macrophage cell line (NR8383) for 15 nm and 55 nm silver particles at the same concentration [49]. However, Li et al. reported a higher O_2_^-•^ formation under UV light for citrate coated particles when compared to PVP coated NPs of similar size [51]. Our study confirms the following ranking with respect to carbonyl induction: Ag 50 PVP > > Ag 50 Citrate = NM-300 k > Ag 200 PVP. Interestingly, ESR data did not reveal a size dependency, as 50 and 200 nm PVP coated silver NPs were rather similar (Ag 200 PVP even had stronger effects when using the CPH probe). Thus, the size dependency that we observed in the carbonylation assay in vitro might be caused by other effects such as different uptake rates.

For the different TiO_2_ variants ESR predicted an activity for NM-103 [rutile] and NM-105 [15 % rutile/85 % anatase]. However, in our biological in vitro test system (NRK-52E cells) we detected an effect only for TiO_2_ NM-105. In general, anatase TiO_2_ NPs are more cytotoxic than rutile NPs [56]. This effect might be caused by photocatalytic activity and has also been demonstrated by Yin et al. who compared anatase particles with rutile and mixed-structure particles [57]. Under UV irradiation they found that the NPs with mixed anatase/rutile structure showed the highest toxicity in HaCaT cells followed by anatase particles, while rutile TiO_2_ showed the lowest toxicity [57].

Both ZnO variants (NM-110, NM-111) were classified as positive in our protein carbonylation study and were highly cytotoxic. ZnO NM-111, which is a coated material, appeared to be slightly less reactive in the 1D carbonyl screening. ZnO NM-110 was tested with two different dispersion protocols. ESR measurements indicated that NM-110 is far less reactive when dispersed by ultrasonication compared to the stirring procedure. This may be due to alteration of the NP surface by ultrasonication, similar to what has been reported for TiO_2_ [58]. However, in biological systems we found that ZnO NM-110 is slightly less reactive, when being dispersed by stirring with respect to carbonylation. Obviously in biological systems the situation is more complex. At present we can only speculate that particles obtained after stirring for 24 h or ultrasonication differ with respect to dissolution of Zn^2+^ ions, agglomeration stability and/or corona formation. All of which will influence uptake rate and cell death. Note for instance that dispersions via ultrasonication are achieved in a significantly shorter time frame. Therefore, both protocols may have distinct effects on the formation of a protein corona. As both techniques have different energy intakes they may also result in different ZnO dissolution rates.

For none of the ZrO_2_ particles, BaSO_4_ NM-220 or AlOOH a clear cytotoxic response could be found. MWCNTs elicited a response only in the DCFDA assay. Interestingly, cytotoxicity in case of the MWCNTs was dependent on the kind of cell model chosen. We have also tested MWCNTs in A549 cells and in THP-1 derived macrophages (data not shown). A strong cytotoxic effect and oxidative stress as detected by protein carbonylation was observed in THP-1 macrophages for NM-401 (MWCNT with the highest aspect ratio). We did not detect cytotoxicity of MWCNT in NRK-52E or A549 cells. BaSO_4_ and AlOOH may be considered as negative benchmark materials.

## Conclusions

Our study employed three different approaches to analyze the oxidative stress potential of a panel of 24 different NPs. Cell-free ESR spectroscopy and in vitro analysis of carbonyl patterns appear to be well suited for this purpose while the use of the DCFDA assay is limited due to NP interferences.

The analysis of protein carbonylation appears to be a very sensitive method to detect possible adverse effects of NPs. Furthermore, we observed a good correlation between ESR and carbonyl results, suggesting that most NPs used in our study induce oxidative stress mostly due to surface reactivity. In addition, we found a good correlation between the results obtained in the carbonyl assay and the overall NP toxicity as tested by WST-1 assay. Thus, we propose that the analysis of protein carbonylation after 1D immunoblotting can be used as a predictive screening method to identify NPs of concern. An advantage of this approach is that in a second step the identification of specifically altered proteins is possible via 2D separation. The more detailed proteomic analysis appears to be a promising tool to unravel underlying toxicity mechanisms. Hence, redox profiling might prove useful for NP classification according to their mode of action.

## Methods

### Nanoparticles

Of the 24 NPs used in this study, 15 NPs were provided through the BMBF funded project nanoGEM, eleven of those were specifically synthesized for the nanoGEM project to introduce different surface modifications on three different core materials (i.e. silica, zirconia and silver, Table 1). The SiO_2_ variants (SiO_2_ unmodified, SiO_2_ Amino, SiO_2_ Phosphate and SiO_2_ PEG), all 15 nm in nominal size, were obtained from BASF. ZrO_2_ (ZrO_2_ Acryl, ZrO_2_ TODS, ZrO_2_ Amino and ZrO_2_ PEG), all 10 nm in nominal size, were obtained from CeraNovis (Germany) and 50 nm as well as 200 nm silver NPs (Ag 50 PVP, Ag 50 Citrate, Ag 200 PVP) from Bayer Technology Services (Germany). These 11 NPs were synthesized by sol–gel or precipitation and were available in aqueous suspensions. In addition, we used boehmite (AlOOH) from Bayer Materials Science (Germany), BaSO_4_ NM-220 from Solvay (Germany), TiO_2_ NM-105 and ZnO NM-110 from the JRC repository (Ispra, Italy), all of which were obtained as powders. A complete characterization data set is available on the nanoGEM webpage.(http://www.nanogem.de/cms/nanogem/upload/Veroeffentlichungen/nanoGEM_Del1.3.1_Characterization_Materials_2013_04_24.pdf).

Additionally, 10 NPs from the EU FP7 project MARINA were used in this study, all of which were obtained from the JRC repository (Ispra, Italy), mostly as powders. These are ZnO (NM-110, NM-111), TiO_2_ (NM-103, NM-104), Ag NM-300 K, and MWCNT (NM-400, NM-401, NM-402). Extensive characterization reports for these NPs are provided by the JRC ([59–63]).

Similarly, the JRC NM nanomaterials differ in surface modification and size or aspect ratio (refer to Table 1). For instance TiO_2_ NM-103 and NM-104 have rutile structure and are stabilized with polydimethylsiloxane while NM-105 is a mixture of rutil and anatase and is unmodified. For ZnO, NM-110 is uncoated, while NM-111 is triethoxycaprylsilane stabilized. The MWCNTs differ mainly in their aspect ratio, with NM-401 being the longest, followed by NM-402.

### NP dispersion

NPs were dispersed using different protocols for reasons as follows. For all nanoGEM NPs a stirring-based protocol was applied. These NPs were hydrophilic and mostly carried chemical surface modifications, which might be affected by ultrasonication. A stock dispersion (2.5 mg/mL) in sterile complete cell culture medium (CCM) consisting of DMEM medium (w/o phenol red and L-glutamine, PAN Biotech GmbH) supplemented with 10 % non-heat inactivated fetal calf serum (FCS gold, PAA Laboratories, GmbH, Germany), 25 mM HEPES buffer (PAN), 100 IU penicillin (PAN), 0.1 mg/mL streptomycin (PAN) and 2 mM L-glutamine (PAN) was prepared by stirring in sterile vials with a magnetic stir bar at 700 rpm for 24 h. Final working dilutions (between 5 and 100 μg/mL) were prepared by diluting the stock dispersion and stirring for another 1 h prior to treatment of cells. Dispersions were prepared freshly for each experiment. We used non-heat inactivated serum, which was considered to be more relevant for comparison to physiological situations.

MARINA NPs were dispersed using sonication-based protocols because part of the NPs were hydrophobic and could not be dispersed well by the stirring-based protocol as determined in preliminary dispersion tests. Hydrophilic NPs (SiO_2_ NM-200/203, Ag NM-300 k, TiO_2_ NM-104, ZnO NM-110) were dispersed at 1 mg/ml in ddH_2_O by sonication for 6 h in an ultrasonic bath (primary dispersion) (Bandelin, Germany). Hydrophobic NPs (TiO_2_ NM-103, ZnO NM-111) were pre-wetted with ethanol and then dispersed in ddH_2_O containing 0.05 % BSA at 2.56 mg/ml by probe sonication with 15 % amplitude for 15 min (Bandelin, Germany). Note, that for the DCFDA assay all MARINA NPs were dispersed according to the protocol for hydrophobic NPs. MWCNTs were dispersed at 1 mg/mL in 1 % (w/v) pluronic in ddH_2_O by first stirring for 30 min and subsequent probe-sonication for 90 min as described elsewhere [64]. Dispersions were stored, but prior to treatment NPs were re-dispersed by sonication for 30 min. Only after re-dispersion were NPs added to CCM.

### Characterization of NPs

#### Dynamic Light Scattering (DLS) and zeta potential

Sizes and size distribution in different dispersion media were measured using a Zetasizer NanoZS (Malvern, Germany) equipped with a He-Ne laser (633 nm). Settings of attenuator and voltage were selected automatically. For DLS measurements dispersions with a concentration of 50 – 100 μg/mL were used. Three independent experiments were performed, comprising of five measurements each.

#### Surface reactivity analysis by ESR

ROS activity of NPs was determined by ESR spectroscopy using two different methods. Employing the method of Papageorgiou et al., that uses CPH (1-hydroxy-3-carboxy-pyrrolidine) as spin probe, possible (surface) reactivity was investigated [65]. Additionally, employing the method of Shi et al. [66], potential hydroxyl radical (OH•) formation was determined in the presence of hydrogen peroxide (H_2_O_2_) and the spin trap 5,5-dimethyl-1-pyrroline-N-oxide (DMPO). The surface reactivity was calculated as ratio between radical formation in the presence of the NPs and the response of deionized water (dH_2_O) as reference signal (Table 2). The suspensions were used as delivered by the provider and have been diluted by a factor of four (for DMPO analysis) or two (for CPH analysis), respectively. For the solid materials we prepared a suspension of 1 mg/mL meaning that a final concentration of 0.25 mg/mL (DMPO) and 0.5 mg/mL (CPH), respectively, was analyzed. Supernatants were also used as delivered by the provider. For the production of the supernatant ultracentrifugation has been performed by providers themselves.

Based on ESR data for BaSO_4_ NM-220, which we considered being a negative reference NP showing no biological responses [67, 68], we defined ESR ratios of >2.6 as positive. ESR values for BaSO_4_ NM-220 were determined to be 2 and we included a 30 % uncertainty resulting from the method. For some selected NPs we also measured NP-depleted supernatants. For those we compared the NP dispersion to the NP-depleted controls and a difference of >2.6 was considered positive. These assessment factors are specific to our system and serve as a guideline only. There are no absolute assessment criteria for ESR being published. Initial tests showed no significant differences in the ROS activity of selected materials after different dispersion methods, e.g. rigorous mixing on a Vortex for 1 min, or stirring for 1 h or 24 h. Consequently, 1 min rigorous mixing on a Vortex was used as dispersion procedure prior to ESR measurements.

Based on previous work using BCR 723 Road Dust no significant interferences with suspended particles were expected. Note that ESR method is not an optical method, which are usually more susceptible to interferences.

### Cell culture

NRK-52E cells (DSMZ, Braunschweig, Germany) were grown in complete cell culture medium (CCM) consisting of DMEM medium (w/o phenol red and L-glutamine, high glucose, PAN Biotech GmbH) supplemented with 10 % non-heat inactivated fetal calf serum (FCS gold, PAA Laboratories, GmbH, Germany), 25 mM HEPES buffer (PAN), 100 IU penicillin (PAN), 0.1 mg/mL streptomycin (PAN) and 2 mM L-glutamine (PAN). Note that ten different cell lines were tested initially and among them NRK-52E was the most responsive epithelial cell line to induce NP-mediated oxidative stress.

Cells were split regularly at ~95 % confluence. For analyzing cell viability with WST-1 assay cells were seeded at 1.25 × 10^4^ cells/well in 96-well plates. For the DCFDA assay cells were seeded in 96-well plates at a density of 3 × 10^4^ cells/well. For the protein carbonyl assays, cells were seeded at 1.5 × 10^6^ cells/well in 6-well plates (1D screen) and for the 2D-based proteomic study cells were treated identically but lysates of 6 wells were pooled for every treatment.

### WST-1 assay

Cells were treated in 96-well plates for 24 h with NPs in concentrations ranging from 0 to 100 μg/ml (0–30 μg/cm^2^). WST-1 reagent was obtained from Roche (Germany) and used according to manufacturer instructions. Before read-out, supernatants were centrifuged at 18,500 × g for 25 min to remove interfering NPs and then measured at 450 nm using a Tecan® (Austria) plate reader. For MWCNTs the centrifugation time was extended to 45 min. All NPs were tested for interference with the assay. To that end we incubated NPs with fully reacted WST-1 reagent (but without cells) and centrifuged as described above to assure that there were no NP interferences.

NP concentrations for in vitro tests were chosen to correlate to relevant in vivo test concentrations. Inhalation overload dose should correspond to 1–10 μg/cm^2^ in in vitro testing, rendering this concentration range the preferred test concentration for in vitro testing. Higher doses were tested in addition for completeness.

### DCFDA assay

The DCFDA assay was performed as described previously [69]. Briefly, 24 h after plating, cells were exposed to NPs for 1 h in a concentration range from 3.4 μg/mL to 34 μg/mL (1–10 μg/cm^2^). For most NPs higher NP concentrations could not be tested due to the onset of NP interferences, which had been evaluated beforehand. ZnO, TiO_2_, NM-200 and NM-203 and MWCNTs interfered less and could be tested at concentrations of up to 170 μg/ml (50 μg/cm^2^). Subsequently, cells were washed twice with Krebs-Ringer Buffer (KRB), incubated with H_2_DCFDA-DA (5 μM in KRB) for 45 min, and washed again twice before monitoring the fluorescence signal (excitation 485 nm, emission 520 nm; FLUOstar, BMG Labtech GmbH, Offenburg, Germany). Each experiment was repeated at least 3 times with 6 or 7 replicates each. To test for interference we incubated NP dispersions (1–100 μg/cm^2^) in the presence of oxidized fluorescent DCF, both with and without cells present. Concentrations with significant changes from the control were excluded from testing.

### Analysis of protein carbonyls via 1D immunoblot

In vitro samples: NRK-52E cells were treated with NP concentrations between 5 and 100 μg/ml (1.7-34 μg/cm^2^) for 6 h (using four different concentrations per NP, mostly below cytotoxic level). Optimal treatment times were determined at the beginning of our study by testing different time points (Additional file 1: Figure S1). Where applicable, NP-depleted supernatants served as a control in addition to untreated cells. Cells were lysed in a modified RIPA-Buffer (50 mM Tris/HCl pH 7.4, 150 mM NaCl, 1 mM EDTA, 1 % Igepal, 0.25 % Na-deoxycholate) containing protease inhibitors (Protease Inhibitor Cocktail Set III, Millipore, Germany) and 2-mercaptoethanol (1 % v/v) at 4 °C for 45 min, followed by centrifugation (4 °C, 17,000 × g, 15 min). Each analysis was performed using at least three independent biological repeats.

In vivo samples: Rat lung tissue was kept at −80 °C after explantation. The lung tissue was pulverized to a fine powder at ~ −180 °C (under Liquid Nitrogen) using a mortar and a pestle and subsequently extracted using RIPA buffer as described above. Samples of four animals per treatment were analyzed.

In all samples protein concentrations were determined using BioRad® Bradford assay (BioRad, Germany) according to manufacturer instructions. Protein carbonyls were detected using the Millipore Oxiblot® kit (Millipore, Germany) according to manufacturer instructions using 10–25 μg protein lysates. Briefly, proteins were separated by 10 % SDS-PAGE and transferred to nitrocellulose membranes using a semi-dry transfer (200 mW/Gel, 1 h). Membranes were blocked with Rotiblock® (Carl-Roth, Germany), and incubated with primary antibodies (1:300 in Rotiblock, from the Oxiblot® kit) overnight at 4 °C. Membranes were washed three times using TBST, incubated with secondary antibodies (1:150 in TBST, from the Oxiblot® kit) for 1 h, washed again 3 times with TBST and visualized using ECL (Thermo Scientific Pierce, SuperSignal West Pico ECL). Chemiluminescence was detected using a GelDoc (BioRad, Germany). Analysis was performed with ImageLab software (BioRad, Germany). For normalization the tubulin signal of the same membrane was used. The tubulin antibody was obtained from Abcam (via NEB, Germany), and used in a 1:5000 dilution in TBST.

### Analysis of protein carbonyls via 2D immunoblot

Cells were treated with 10 μg/ml (3.4 μg/cm^2^) NP for 6 h and lysed in 600 μl 2D lysis buffer (7 M urea, 2 M thiourea, 4 % Chaps, 2 % IPG buffer pH 3 –10, 1 % DTT) at 4 °C for 45 min. 350 μg of the protein lysate were used for every analysis. For mass spectrometric identification we used a separate duplicate 2D gel. For isoelectric focusing (IEF), 24 cm IPG strips (GE Healthcare, Germany) with a nonlinear pH gradient of pH 3 –10 were used. Active rehydration and focusing was performed in 6 steps (15 h at 30 V, 1.5 h at 200 V, 1 h at 500 V, a 13.5 h gradient from 500 –1000 V, a 3 h gradient from 1000 –8000 V, and 6 h at 8000 V). After IEF, protein carbonyls were derivatized in the IEF strip by incubation with 10 mM 2,4-dinitrophenylhydrazine (DNPH) in 2 M HCl at RT for 15 min. Excessive reagent was removed by three washes of 2 M tris (hydroxymethyl) aminomethane solution in 30 % glycerol/ddH_2_O for 10 min each. Subsequently, strips were washed twice with electrophoresis buffer for 10 min. Samples were reduced with DTT and alkylated using iodoacetamide according to standard protocols prior to separation in the second dimension. For second dimension separation 12.5 % SDS-PAGE gels were used with 3 W per gel for 1.5 h followed by 15 W per gel. Proteins were transferred onto a nitrocellulose membrane using wet-transfer (50 mA, 16 h). Membranes were blocked with Rotiblock® (Carl-Roth, Germany). Carbonyls were detected using DNBP antibody (Sigma, Germany) at 1:500 in Rotiblock® at 4 °C over night. After three washes, a goat anti-rabbit antibody (Dianova, Germany) was used at 1:10,000 in TBST for 2 h at room temperature. For visualization by ECL a GelDoc imager (BioRad) was used. In parallel, each sample was separated on another duplicate 2D gel, which was stained with ruthenium II-bathophenanthroline disulfonate chelate as described elsewhere [70]. Gels were scanned using a FLA9500 laser scanner (GE Healthcare, Germany), using an excitation at λ = 473 nm and detection at λ = 610 nm. Duplicate gels were used for spot picking and mass spectrometric identification.

Analysis of 2D gels and 2D immunoblots was performed using Delta 2D software (Decodon, Germany). A carbonyl spot was considered relevant if it was detected in 2 out of 3 biological repeats (for NP samples) or in at least 4 out of 6 repeats for control gels. All NP samples were analyzed in at least three independent biological replicates, controls in 6 independent biological replicates.

### Identification of protein by MALDI-TOF/TOF

Spots were excised from the fluorescent stained gels using a spot picker (Proteome Factory, Germany) and digested in-gel with trypsin using a standard protocol. In short, gel spots were incubated with 0.03 μg trypsin in 50 mM ammonium bicarbonate in 95/5 H_2_O/acetonitrile over night at 37 °C for digestion. Peptides were extracted from the gel matrix by subsequent extraction with 60 % acetonitrile (ACN)/0.1 % trifluoroacetic acid and 100 % ACN. Digested samples were dried in a centrifugal evaporator, redispersed in 1 % TFA and purified using a C18 ZipTip (Millipore, Germany). Samples were spotted with α-cyano-4-hydroxy-cinnamic acid (HCCA) matrix on AnchorChip targets (384/800) (Bruker, Germany) and measured using an UltrafleXtreme MALDI-TOF/TOF (Bruker, Germany) with FlexControl and FlexAnalysis software. Data were evaluated using Proteinscape (via MASCOT using Swissprot database). The following search parameters were used: 1 partial cleavage site, carbamidomethyl (Cys) as fixed modification and oxidation (Met) as variable modifications. Taxonomy was *rattus*, MS tolerance was 50 ppm and MS/MS tolerance was 0.7 Da. Proteins were considered reliably identified if the MASCOT score was above threshold and in addition at least 2 independent unique MS/MS spectra were identified. Proteins identified by a MASCOT score with only 1 unique MS/MS spectrum were considered as not reliably identified and those were marked as ‘tbc’ (Additional file 1: Table S1).

### Pathway analysis

For pathway analyses we used Ingenuity Pathway Analysis software (IPA^TM^, Qiagen, Germany). Data sets of identified proteins were subjected to core analyses against the IPA knowledge base (genes only data base version) for protein enrichment in canonical pathways.

### Statistical analysis

For each spot in each treatment median values were derived from the spot intensities of the 2D carbonyl immunoblots as obtained by Delta 2D analysis. Median values of all carbonyl spots (202 spots) were analyzed using the statistical computing environment R [R Development Core Team, 2012]. Some spots appeared only in the treated samples and were absent in control samples. If spots appeared both in treated samples as well as in controls, spots were considered for analysis that were significantly induced compared to controls (intensities at least 2-fold over controls, *p* < 0.05, students t-test, in total 175 spots). We performed a hierarchical cluster analysis (HCA) using Euclidian complete linkage. The basic idea of HCA is to group a given data set step by step into nested clusters by using a distance measure. Here we used the spot numbers of carbonylated proteins together with median intensities resulting from minimum three biological repeats to analyze distances between the different NP treatment groups. We employed Euclidian complete linkage as one of the many possible methods, which uses a Euclidian distance measure and always uses the largest possible distance between two clusters of data points for analysis.

In addition we performed principal component analysis (PCA) using SIMCA software (Umetrics, Sweden) using 5 main principal components, which could explain more than 65 % of data variance. The objective of PCA is to project data points from a higher into a lower dimensional space with minimal loss of information. It visualizes variance in a data set by introducing new axes (called principle components) such that the first axis lies in the direction of greatest variation. Each subsequent principal component (PC) describes the largest variance in a direction orthogonally to the previous PC, thus resulting in linearly uncorrelated PCs.

### Animal experiments

Pathogen-free female rats (Wistar strain WU) weighing 200–250 g were purchased from Charles River Laboratories (Sulzfeld, Germany) and maintained with a 12 h lights-on lights-off cycle. Food and water were provided ad libitum. For intratracheal instillation animals were briefly anesthetized using isoflurane. Animal experiments were approved by the local regulatory agencies. All study protocols complied with the federal guidelines. An Ag 50 PVP stock dispersion was diluted in 0.9 % NaCl (which also served as control treatment without NPs) and indicated doses were intratracheally instilled using a Penn Century Microsprayer that had been inserted into the trachea under visual control. On the day of sacrifice, rats were deeply anesthetized with a mixture of ketamine and xylazine. The trachea was cannulated and the left lung filled with Cryomatrix (Thermo Shandon Ltd., Runcorn, UK): A tissue block (0.5 – 1 cm^3^) from the medial hilar region was cut with razor blade, snap frozen in liquid nitrogen, and stored at −80 °C.

## Additional file

Additional file 1: Figure S1-S5
**and** **Table S1.** The file contains various additional figures and tables, collected in a single portable documend file (PDF) (DOC 9935 kb).
