# Supplementary material for: Proteomic analysis of protein carbonylation: a useful tool to unravel nanoparticle toxicity mechanisms
Source: Part Fibre Toxicol. 2015 Nov 2;12:36. doi: 10.1186/s12989-015-0108-2 (PMC4630844; doi:10.1186/s12989-015-0108-2)
Supplement: Additional file 1: Figure S1-S5 — and Table S1. The file contains various additional figures and tables, collected in a single portable documend file (PDF) (DOC 9935 kb). [file 12989_2015_108_MOESM1_ESM.doc]

Supporting Information

Proteomic analysis of protein carbonylation: A useful tool to unravel nanoparticle toxicity mechanisms

Marc D. Driessen^1^, Sarah Mues^2^, Antje Vennemann^3^, Bryan Hellack^4^, Anne Bannuscher^1^, Vishalini Vimalakanthan^1,6^, Christian Riebeling^1^, Rainer Ossig^2^, Martin Wiemann^3^, Jürgen Schnekenburger^2^, Thomas A. J. Kuhlbusch^4,5^, Bernhard Renard^6^, Andreas Luch^1^ and Andrea Haase^1*^

^1^German Federal Institute for Risk Assessment (BfR), Department of Chemicals and Product Safety, Berlin, Germany,

^2^Biomedical Technology Center, Westfälische Wilhelms-University, Münster, Germany,

^3^IBE R&D gGmbH, Institute for Lung Health, Münster, Germany,

^4^Institute of Energy and Environmental Technology (IUTA) e.V., Air Quality & Sustainable Nanotechnology, Duisburg, Germany,

^5^Center for Nanointegration CENIDE, University of Duisburg-Essen, Duisburg, Germany,

^6^Robert-Koch-Institut (RKI), Junior Research Group Bioinformatics, Berlin, Germany.

*Corresponding author


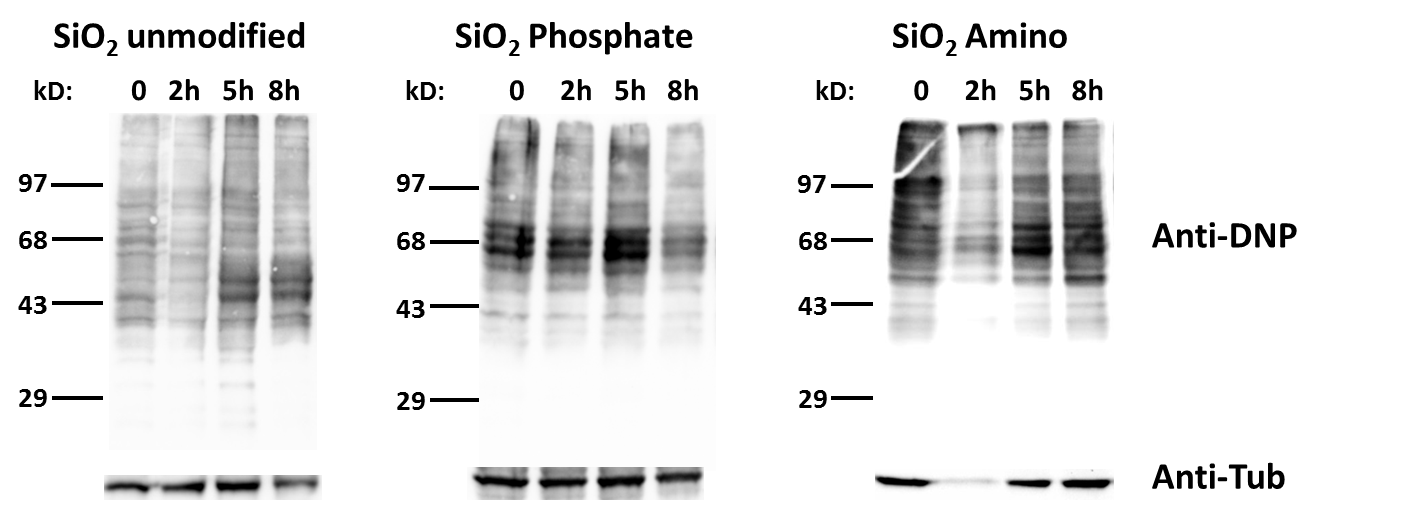


Figure S1: Kinetics of protein carbonylation in NRK-52E cells for different SiO_2_ NPs

NRK-52E cells were incubated with 10 µg/mL (3.4 µg/cm^2^) for the indicated time period (0, 2h, 5h, 8h). Carbonylation was detected in the cellular lysates after coupling the modified proteins with dinitrophenyl-hydrazine (DNPH) using a specific DNP antibody in immunoblots. Normalization was performed with tubulin signals. All experiments were performed in at least three independent biological repeats. A typical result for each NP is depicted here.


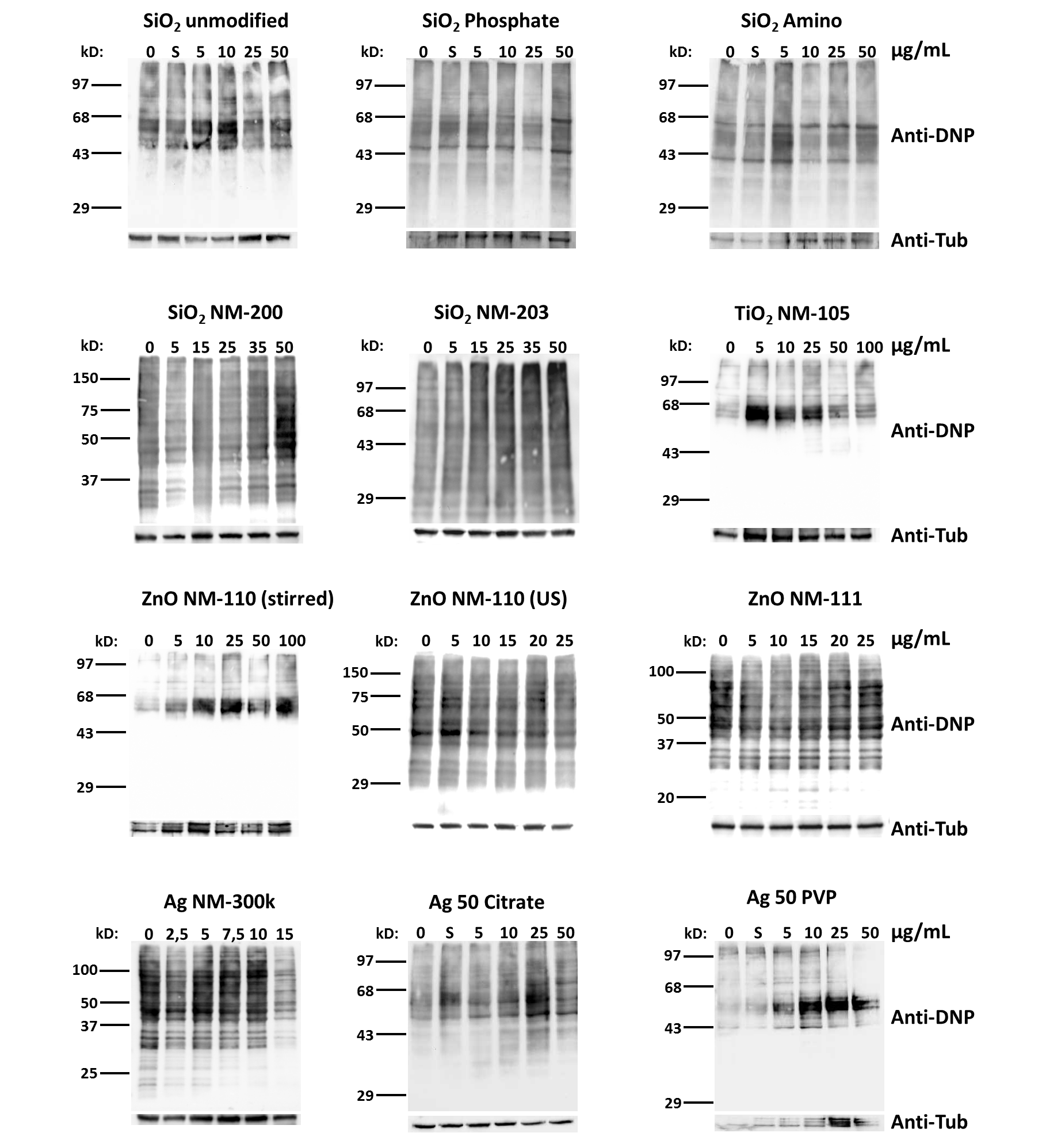


Figure S2: Immunoblots of all positive NPs for protein carbonylation in NRK-52E cells.

NRK-52E cells were incubated with the respective NP in the indicated concentration (0 to up to 100 μg/ml (34 µg/cm^2^), in case of strong cytotoxicity the concentration was adjusted accordingly) for 6h. Carbonylation was detected in the cellular lysates after coupling the modified proteins with dinitrophenyl-hydrazine (DNPH) using a specific DNP antibody in immunoblots. Normalization was performed with tubulin signals. All experiments were performed in at least three independent biological repeats. A typical result for each NP is depicted here.

Figure S3: Immunoblots of all negative NPs for protein carbonylation in NRK-52E cells.

NRK-52E cells were incubated with the respective NP in the indicated concentration (0 to up to 100 μg/ml (34 µg/cm^2^)) for 6h. Carbonylation was detected in the cellular lysates after coupling the modified proteins with dinitrophenyl-hydrazine (DNPH) using a specific DNP antibody in immunoblots. Normalization was performed with tubulin signals. All experiments were performed in at least three independent biological repeats. A typical result for each NP is depicted here.


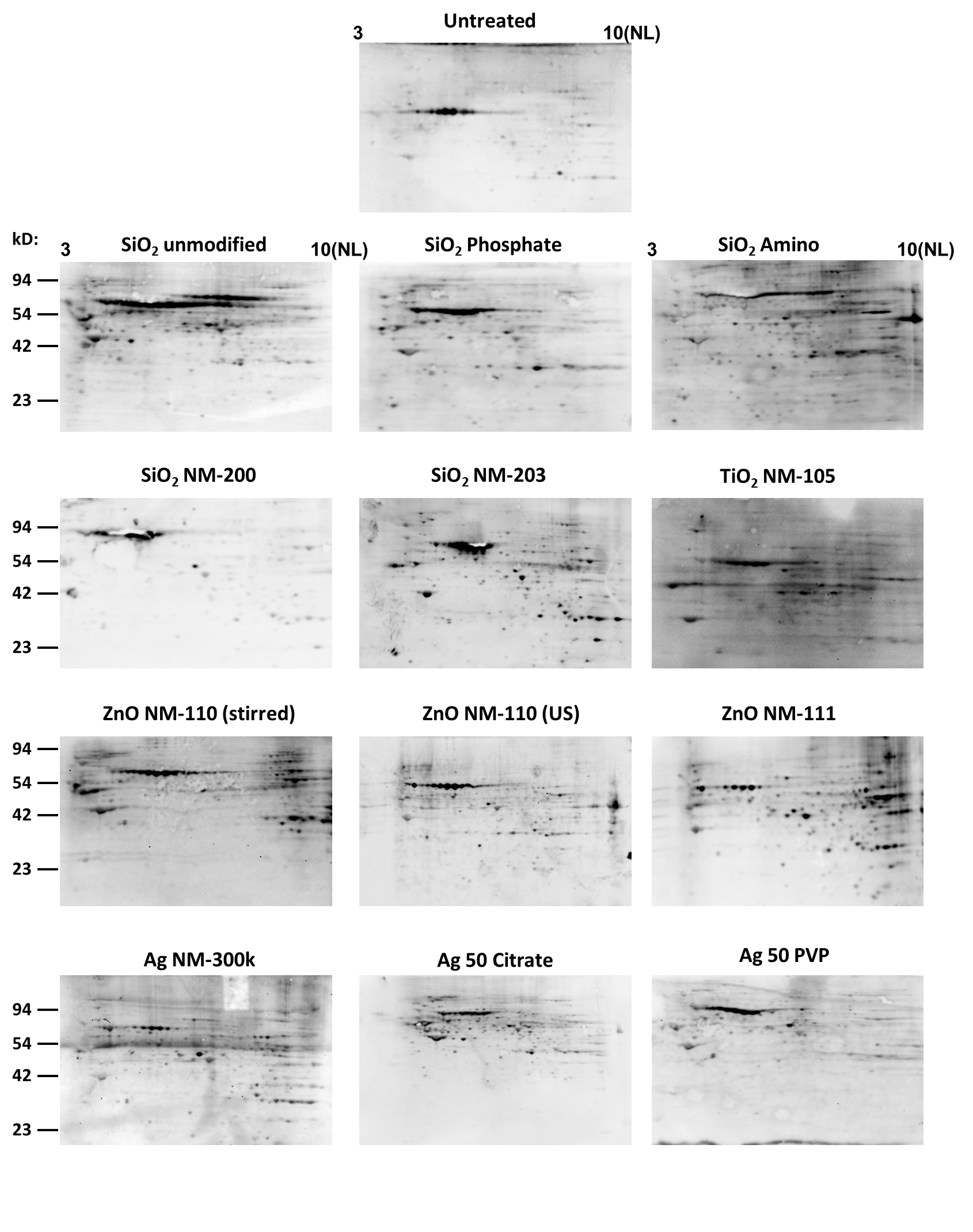


Figure S4: 2D immunoblots for all positive NPs

NRK-52E cells were incubated with the respective NP at 10 μg/ml (3.4 µg/cm^2^). Carbonylated proteins were labeled by using dinitrophenyl-hydrazine (DNPH) after separation in first dimension. Labeled proteins were detected in 2D immunoblots using an anti-DNP antibody. All experiments were performed in at least three independent biological repeats. A typical result for each NP is depicted here.


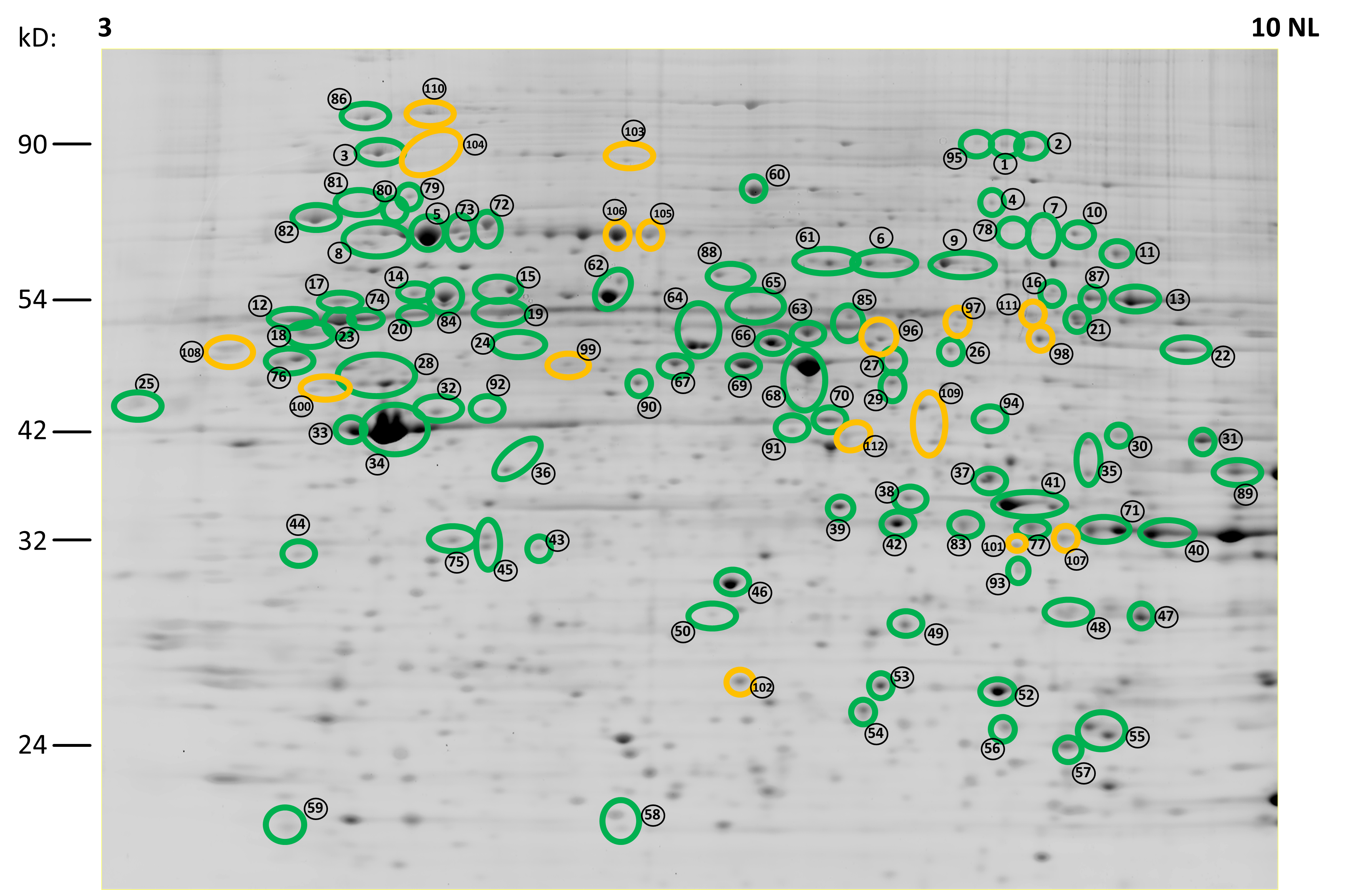


Figure S5: Identified modified proteins

Depicted is one corresponding duplicate gel image of a untreated control sample, which were run in parallel to the 2D immunoblots shown in Supplementary Figure S4 visualizing all cellular proteins not only the modified ones of the 2D immunoblots. Modified proteins as identified in 2D immunoblots were excised from the duplicate gels and identified by mass spectrometry. All proteins that could be identified are marked in this image with green marks. Yellow labels (roman numbers) refer to proteins, which are identified only on basis of the Mascot score and 1 MS/MS (i.e. likely identified). Protein identities are given in Table S1.

**Table S1. Proteomic identification assigned to the respective NP treatment.** Column 1 refers to the spot number (Figure S5). Column 2 gives additional information. Single means the respective spot could be clearly identified on the duplicate gel by transferring the spot from the 2D blot, multiple means that several spots had to be considered (see Materials and Methods). Columns 3 - 8 list identification parameters. Each of the following columns refers to a NP treatment while x means the spot had similar intensity after NP treatment compared to control, xx and xxx indicating stronger intensities than in control).
